# G-Net: Implementing an enhanced brain tumor segmentation framework using semantic segmentation design

**DOI:** 10.1371/journal.pone.0308236

**Published:** 2024-08-06

**Authors:** Chandra Sekaran D. S., Christopher Clement J.

**Affiliations:** School of Electronics Engineering, Vellore Institute of Technology, Vellore, Tamilnadu, India; Shijiazhuang Tiedao University, CHINA

## Abstract

A fundamental computer vision task called semantic segmentation has significant uses in the understanding of medical pictures, including the segmentation of tumors in the brain. The G-Shaped Net architecture appears in this context as an innovative and promising design that combines components from many models to attain improved accuracy and efficiency. In order to improve efficiency, the G-Shaped Net architecture synergistically incorporates four fundamental components: the Self-Attention, Squeeze Excitation, Fusion, and Spatial Pyramid Pooling block structures. These factors work together to improve the precision and effectiveness of brain tumor segmentation. Self-Attention, a crucial component of G-Shaped architecture, gives the model the ability to concentrate on the image’s most informative areas, enabling accurate localization of tumor boundaries. By adjusting channel-wise feature maps, Squeeze Excitation completes this by improving the model’s capacity to capture fine-grained information in the medical pictures. Since the G-Shaped model’s Spatial Pyramid Pooling component provides multi-scale contextual information, the model is capable of handling tumors of various sizes and complexity levels. Additionally, the Fusion block architectures combine characteristics from many sources, enabling a thorough comprehension of the image and improving the segmentation outcomes. The G-Shaped Net architecture is an asset for medical imaging and diagnostics and represents a substantial development in semantic segmentation, which is needed more and more for accurate brain tumor segmentation.

## 1 Introduction

Brain Tumor Segmentation (BTS) is a medical computational image analysis technique pertaining to identifying brain tumors from healthy brain tissues in magnetic resonance imaging (MRI) data. The ultimate goal of focusing on BTS is to develop a multidimensional segmentation model that properly portrays the tumor’s precise position and size. Glial cells in the brain and spinal cord are the source of the common malignant brain tumor known as glioma. The median survival time for glioma patients is about 12 months, and gliomas are aggressive cancers. The need of early tumor detection makes MRI a key tool in this process. The numerous sequences of MRI, such as T1-weighted, T2-weighted, T1-weighted contrast-enhanced, and T2 Fluid Attenuated Inversion Recovery, which emphasize various tumor characteristics, give high spatial resolution anatomical information. For the purpose of tumor diagnosis, the annotation and segmentation of tumor borders must be accurate. Manual segmentation, on the other hand, is expensive, time-consuming, and prone to human mistake, particularly when tumors have diverse intensities and forms in various sub-regions. The identification of brain anomalies on MR imaging involves various kinds of steps, including image preprocessing, feature extraction, characteristics, image enhancement and visualization, segmentation, and classification [1]. M. Ghaffari, A. Sowmya and R. Oliver review the evolution of automated models for BTS using multimodal MR images, highlighting the challenge of developing such methods due to the heterogeneity of brain tumors [2]. Montaha, Sidratul, et al. presented a method for automated BTS from 3D MRI scans using an optimized U-net model. After normalization and rescaling, the model achieves an accuracy of 99.41% and a dice similarity coefficient of 93% [3]. Sangui, Smarta, et al. developed a modified U-Net architecture for detecting and segmenting brain tumors from MRI images using a deep-learning framework. The model attained a test accuracy of 99.4% using BRATS 2020 datasets. The U-Net model outperforms other DL-based models, making it a valuable tool for detecting and analyzing brain tumors compared to other methods [4]. A modified U-Net structure using residual networks and sub-pixel convolution was proposed, enhancing modelling capability and avoiding de-convolution overlapping. The model was evaluated on the Brain Tumor Segmentation (BraTS) Challenge 2017–2018, achieving segmentation accuracy of 93.40% and 92.20%, respectively [5].

Abdullah Al Nasim, M. D., et al. used U-Net to segment brain tumors from MRI images, focusing on necrotic, edematous, growing, and healthy tissue. The 2D U-Net network was trained using the BraTS datasets, reducing computational time by excluding background details. Experiments show the proposed model works well, with dice scores of 0.8717 for necrotic, 0.9506 for edema, and 0.9427 for enhancing [6]. Throughout the world, brain tumors are a common type of tumor. Due to poor and lagging diagnostic techniques and algorithms, this disease is increasingly the main cause of death nowadays. The demands of larger datasets are less effectively satisfied by the brain tumor diagnosis techniques now in use. Larger datasets take longer to process, which slows down system performance. The accuracy, specificity, and sensitivity of the models are evaluated in order to determine how well they work. The G-architecture is designed and developed as a key solution for addressing these significant challenges. The models’ accuracy, specificity, and sensitivity are used to evaluate their performance in the workflow technique.

## 2 Related works

The research focuses on improving autonomous BTS and classification techniques for better diagnosis. Surveys have been conducted to explore techniques used in this medical image analysis field. This survey provides a comprehensive overview of proposed techniques like segmenting, suppressing irrelevant regions, salient feature extraction, machine learning, and deep learning. It also covers the technical aspects, strengths, weaknesses, and performance matrix of the proposed architecture model. The aim is to enhance the diagnostic capabilities of physicians and healthcare professionals.

Roy, Sunita, et al. proposed two new CNN-based models, S-Net and SA-Net, for image segmentation in medical imaging, particularly for brain tumors in MRI scans. These models use U-Net as the base architecture and leverage Merge Block and Attention Block concepts [7]. The U-shaped architecture, with Block-R3 applied, outperformed Block-R1 and Block-R2. The proposed AD unit extracted detailed tumor features efficiently, achieving dice scores of 0.90, 0.80, and 0.76 on the BraTS20 dataset [8]. Modeling channel dependencies, and utilizing multi-scale predictive fusion, resulting in superior segmentation performance compared to existing networks [9]. Ali, Tahir Mohammad, et al. described and developed an attention-based convolutional neural network for BTS using the BRATS’20 dataset. The results show a dice similarity coefficient of 0.83, 0.86, and 0.90 for enhancing, core, and whole tumors [10].

Bindu, N. Phani, and P. Narahari Sastry used ResNet50 as an encoder in the U-Net model to enhance segmentation precision and efficacy in medical imaging applications. This approach introduces cross or skip connections between network blocks, reducing the need for frequent skip connections. Test IoU and Test Dice Coeff values of 0.902 and 0.948 were obtained, respectively [11]. A hybrid of the deep residual network and the U-Net model, using the residual network as an encoder and the U-Net model as a decoder to address vanishing gradient issues. Validation on an external cohort showed the model’s robustness in real-world clinical settings, with dice scores of 0.8400, 0.8601, and 0.8221 for TC, WT, and ET, respectively [12]. Vijay, Sanchit, et al. proposed a model called SPP-U-Net, which uses a combination of Spatial Pyramid Pooling (SPP) and Attention blocks to improve performance. The model’s average Dice Score and Haussdorf distance are 0.883 and 7.99, respectively [13]. Baid, U. et al. presented a method for glioma tumor segmentation and survival prediction using a Deep Learning Radiomics Algorithm for Gliomas (DRAG) Model and a 3D patch-based U-Net model. The model achieved good performance in the BraTS-2018, with Dice scores of 0.88, 0.83, and 0.75 for WT, TC, and ET, respectively [14]. Isensee, Fabian, et al. applied an nnU-Net for the BraTS 2020 challenge segmentation task, achieving respectable results. By incorporating BraTS-specific modifications, including postprocessing, region-based training, and data augmentation, the performance is significantly improved. The method achieved and obtained performance with Dice scores of 88.95, 85.06, and 82.03 for the WT, TC, and ET [15].

Biratu, Siyoum, et al. investigated BTS and classification using region-growing, shallow machine learning, and deep learning methods. They discussed usage, pre-processing, feature extraction, segmentation, classification, post-processing, model performance, pros and cons, and model evaluation metrics [16]. For automated BTS, Aboelenein, Nagwa M., et al. suggested a novel MIRAU-Net model incorporating residual inception modules, attention gates, sub-networks encoders and decoders, including a multi-loss function aimed at reducing class imbalance [17]. Yousef, Rammah et al. explored four U-Net architectures (3D, Attention, R2 Attention, modified 3D U-Net) on the BraTS 2020 dataset for BTS, evaluating their performance in terms of Dice score and Hausdorff distance of 95% and emphasizing their significance of visualizations [18].

Major trends for future research strategies are emphasized in the discussion of recent U-Net architecture-based BTS approaches [19]. Yang, Tiejun, and Jikun Song proposed fully automatic brain tumor MRI image segmentation algorithm utilizes a semantic segmentation U-net model, integrating image features from image patch datasets and adding a 1x1 convolutional layer [20]. Prasanna, Gaurav et al. proved a double attention-based scheme for incorporating a squeeze and excitation network and a soft attention mechanism. The model can be tested on a 3D medical imaging dataset to enhance performance and achieve a higher Jaccard coefficient [21].

Automatic BTS for MRI images utilizes encoder-decoder-based convolutional neural networks, particularly UNET and SEGNET [22]. Hmeed, Assef Raad, et al. used the U-net model and a fully convolutional network technique for semantic segmentation at BraTS 2018, achieving mean dice similarity coefficients of 0.87, 0.76, and 0.71 [23]. Agrawal, Pranjal, et al. introduced a 3D U-Net model for volumetric BTS, utilizing a CNN-based automated system for segmentation and feature extraction and a classical neural network for classification [24]. The BRATS benchmark evaluated current methods. Achieving Dice scores of over 80% for the whole tumor, but the active core region is more challenging [25]. Inspired by MobileNetV2 and U-Net, Saeed, Muhammad Usman, et al. developed an effective DL-based RMU-Net model for BTS. It outperforms models with fewer parameters and high dice coefficient scores [26].

DAU-Net is a deep supervised nested segmentation network that employs a modified dense skip connection for feature detection and merging, developed by Na Li and Kai Ren [27]. The SCAU-Net is a 3D U-Net model for BTS, enhancing semantic up-sampling through external attention and self-calibrated convolution, which demonstrated impressive performance on the BraTS 2020 validation dataset with dice similarity coefficients of 0.905, 0.821, and 0.781 [28]. Huang, He, et al. introduced a deep framework for BTS that implements a V-Net-based distance transform decoder for better accuracy and feature extraction. On the 2020 BraTS dataset, the model demonstrated remarkable performance, with Dice metrics of 0.75, 0.86, and 0.77 for the ET, WT, and TC regions, respectively [29].

The work introduces NLCA-VNet, a glioblastoma automated segmentation approach that uses VNet and nonlocal and convolutional block attention modules to improve segmentation performance by retaining more information and performing attention in channel and spatial dimensions [30]. The study introduces AGSE-VNet, an automated brain tumour MRI data segmentation that employs the Squeeze and Excite modules, as well as the Attention Guide Filter, to improve usable information and reduce noise. It confirms Dice scores of WT, TC and ET 0.68, 0.85, and 0.70, respectively [31]. The study describes TransBTS, a network built on an encoder-decoder framework that employs Transformer in 3D CNN for MRI BTS [32].

By extracting information from the entire image, the study suggests modifying U-Net and implementing an attention block (AttU-Net), which raises the Dice score by 5%. The technique was evaluated on the BraTS 2021 challenge dataset and yielded promising ET, TC, and WT scores of 0.793, 0.819, and 0.879, respectively [33]. In this study, a modified Bridged U-Net architecture with Atrous Spatial Pyramid Pooling (ASPP) and an evolving normalisation layer was proposed. The model accomplished both the BraTS 2020 and BraTS 2021 challenge datasets, outperforming other cutting-edge models [34].

Researchers discussed the use of transfer learning for brain tumor classification, highlighting its potential for accuracy improvement. It emphasized the need for large annotated datasets and computational resources. [35] suggested leveraging transfer learning to reduce training time and enhance model performance. The model’s effectiveness was evaluated, revealing its potential for clinical use and enhanced diagnostic accuracy. Techniques were used, including evolutionary algorithms such as genetic algorithms or nature-inspired optimization techniques, and DL techniques for feature extraction and classification. [36] showed promising results in accurately grading and classifying brain tumors, reducing diagnostic errors and improving patient outcomes. The hybrid approach improved quality and robustness, enhancing clinical practice precision in BTS. [37] combined handcrafted features with global pathway-based DL. Handcrafted CNNs are introduced in [38] to improve accuracy and efficiency in BTS from MRI images. Customized to the specific characteristics of the data, this approach shows significant gains above general CNN models. Scalable federated learning is used in [39] to increase accuracy while preserving data privacy and confidentiality, proving the usefulness of these methods in medical image analysis.

CNNs have made tremendous progress recently in improving medical image processing, especially in segmenting and classifying cancer images. [40], when it comes to recognizing cancer cells from breast cytopathology images, CNNs perform better than conventional techniques. Examiner and Mean Teacher models are combined in a new 3D CNN-based semi-supervised learning framework for brain tumor segmentation, which improves segmentation performance by using both labeled and unlabeled data [41]. This research shows how semi-supervised learning methods and CNNs can be used to increase the precision and effectiveness of cancer image analysis.

On these literatures, authors have proposed an automated system for BTS techniques, an architectural design, reproducible segmentation performance similar to manual results. This architecture can alleviate difficulties in manually analyzing brain tumors, speed up image analysis, improve diagnosis outcomes, and facilitate disease follow-up by evaluating tumor progression. In this section, among the proposed BTS techniques in the scientific literature, predictive modeling, ML, neural networks, and DL-based approaches will be reviewed for identifying the clinical dataset, pre-processing, feature extraction, segmentation algorithm, and observed overall outcomes. In order to ensure proper data format, segmentation properties, and accurate labeling for efficient training of deep learning models, this work investigates data preprocessing techniques for medical images with a focus on segmentation tasks.

## 3 Methodology

**Fig 1:**
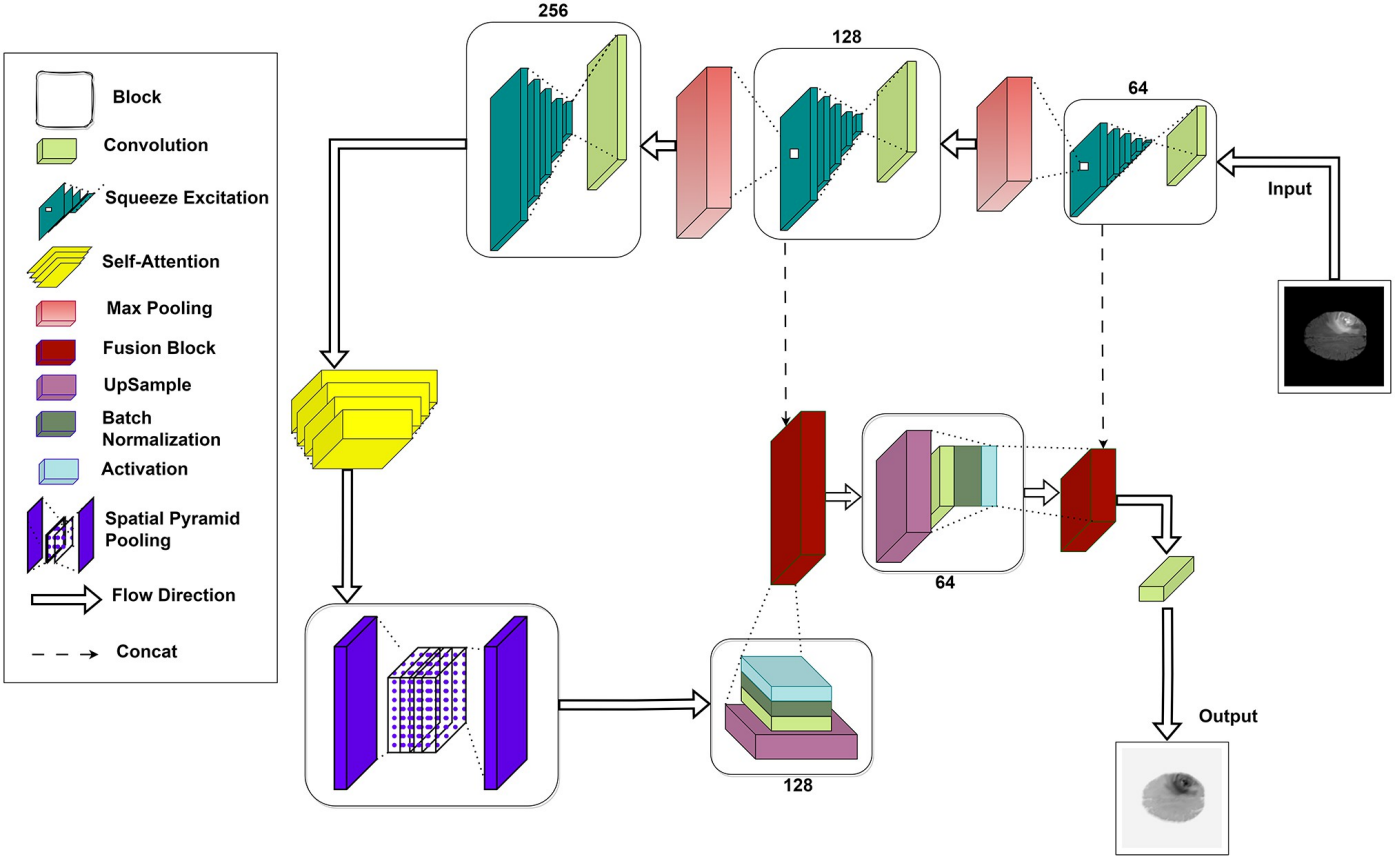
Framework block diagram of the G-Net.

In the methodology section of this research paper, we describe in detail the key building blocks employed in our deep CNN architecture designed for image segmentation tasks. These building blocks, namely convolutional layers, Self-Attention(SA), Squeeze Excitation(SE), Spatial Pyramid Pooling(SPP), and fusion blocks serve to enhance the network’s feature extraction capabilities, attention mechanisms, and multi-scale feature processing, ultimately contributing to improved segmentation performance. Proposed model by integrating convolutional layers, SE, SPP, fusion blocks and SA within the G-Net (G-Shaped Net) architecture as shown in Fig 1. Detailed explanation of each block are given below.

### 3.1 Squeeze excitation block

**Fig 2:**
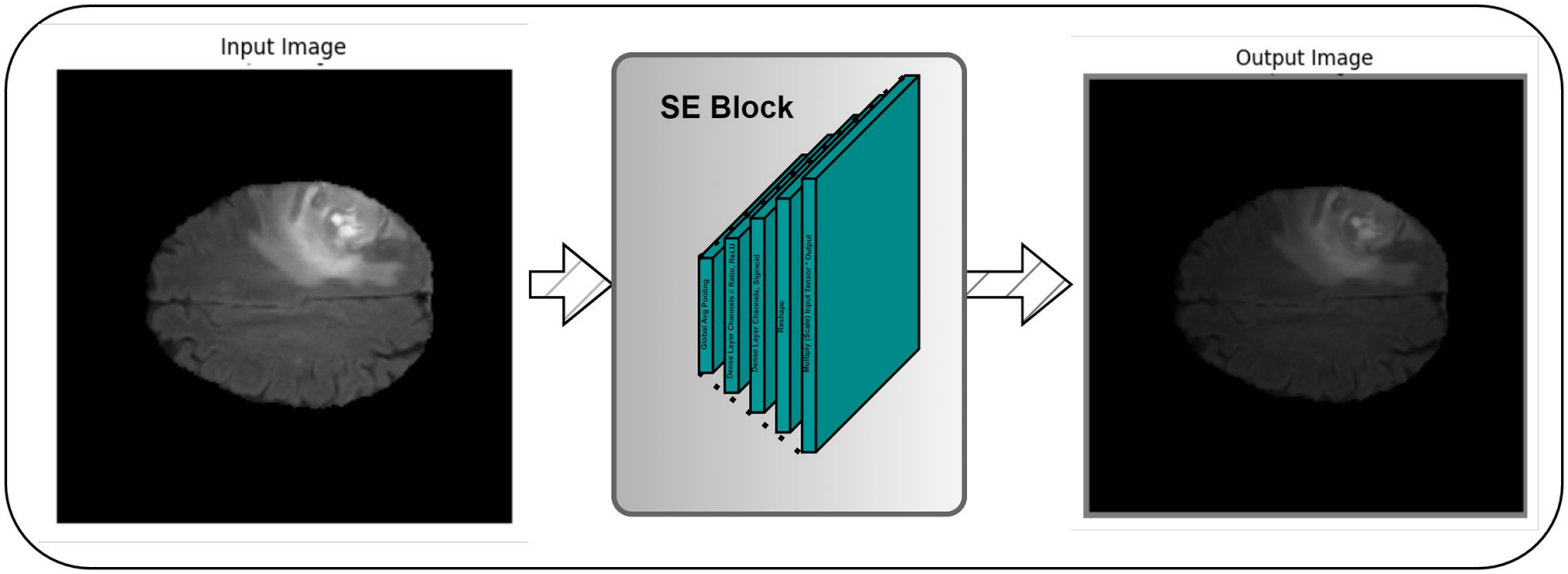
Squeeze Excitation module when a single image passes through.

The Squeeze Excitation (SE) block is a crucial component of the G Net architecture designed to enhance channel-wise feature dependencies in the input tensor. This block begins by applying global average pooling, which computes the average value for each channel across the entire spatial domain of the feature maps. This effectively reduces the spatial dimensions while retaining the channel-wise information. The output from global average pooling is then fed through two fully connected layers with ReLU and sigmoid activations, respectively. These layers adjust the channel-wise weights, allowing the network to emphasize or de-emphasize certain channels based on their importance. Finally, the reshaping operation transforms the result into a tensor with dimensions (1, 1, channels). This tensor is element-wise multiplied with the original input tensor, effectively scaling each channel differently based on learned channel-wise weights. This process helps the network focus on the most relevant features, improving the model’s ability to capture important information for semantic segmentation. Squeeze and Excitation are the two steps that the SE module in our model uses to accomplish feature recalibration. Global Average Pooling is used to aggregate global spatial information during the Squeeze step. This data is utilized in the Excitation stage to create channel-wise dependencies between two fully linked (Dense) layers that have sigmoid and ReLU activations, respectively. The SE module when a input image is given and passed through it and what is the results it reflects after passing through a single SE block is shown in Fig 2.

Mathematical representation of SE block given below:

Given input tensor **X** with dimensions (*H*, *W*, *C*), and considering a single channel:

**Squeeze:** Calculate channel importance for each channel *c* in the input tensor using global average pooling:

$$\begin{array}{c} Z_{s}=\frac{1}{H\cdot W}\sum_{i=1}^{H}\sum_{j=1}^{W}X_{i,j,c} \end{array}$$

*H* represents the height of the input tensor. *W* represents the width of the input tensor. **X**(*i*, *j*, *c*) is the value at spatial coordinates (*i*, *j*) in channel *c* of the input tensor. **Z**_**e**_ and **Z**_**s**_ are matrices representing the channel importance values.

**Excitation:** Refine channel importance using two fully connected (Dense) layers. The first layer introduces non-linearity with ReLU activation, and the second layer squashes values between 0 and 1 with sigmoid activation:

$$\begin{array}{c} Z_{e}=\sigma \left(W_{2}\cdot \text{ReLU}\left(W_{1}\cdot Z_{s}\right)\right) \end{array}$$

**W**_1_ and **W**_2_ are learnable weights. ReLU represents the rectified linear unit activation function. *σ* represents the sigmoid activation function.

Reshape the refined importance values to have the same dimensions as the input tensor (1x1xC). Scale the original input tensor by element-wise multiplication with the importance values:

$$\begin{array}{c} Y\left(i,j,c\right)=X\left(i,j,c\right)\cdot Z_{e} \end{array}$$

$$\begin{array}{c} Y=X⊙Z_{e} \end{array}$$

The result is an output tensor **Y** where each channel’s information is adjusted based on its importance, emphasizing important features and de-emphasizing less important ones. This helps the neural network focus on relevant information in the input data.

### 3.2 Self-Attention block

The Self-Attention (SA) Block introduces an attention mechanism into G-Net, allowing the model to focus on and emphasize specific regions within the input tensor that are deemed most relevant for the task at hand. This block begins by calculating attention weights through a 1x1 convolutional layer with sigmoid activation. These weights are derived from the input tensor and represent the importance of each spatial position within the feature maps. The attention weights are then element-wise multiplied with the original input tensor, resulting in an attention-weighted feature map. The model’s SA mechanism, which softens focus and adapts to input relevance, is particularly useful for tasks like semantic segmentation, ensuring precise localization and feature importance.

The SA Block involves the computation of attention weights using a 2D convolution with a sigmoid activation function:

$$\begin{array}{c} A=\text{Conv2D}(X,(1,1),\text{activation}=\text{Sigmoid}) \end{array}$$

Where, **A** represents the computed attention weights. The (1, 1) kernel size is used for channel-wise attention. Then, input feature map **X** is rescaled using the computed attention weights **A**, resulting in the enhanced feature map **Y**:

$$\begin{array}{c} Y=X⊙A\;\text{Where},⊙\text{represents}\;\text{element-wise}\;\text{multiplication}. \end{array}$$

### 3.2 Spatial Pyramid Pooling block

The Spatial Pyramid Pooling block is a critical feature extraction component within G-Net, designed to capture multi-scale information from the input tensor. This block starts with the original input tensor and constructs a pyramid of features at various spatial resolutions. It does this by repeatedly applying max-pooling, convolution, and up sampling operations with different pooling sizes specified in the pool sizes parameter. Each pooling size captures information at a different scale. After pooling, a 1x1 convolutional layer is used to transform the pooled features, and then they are up sampled to the original size. These feature maps at various scales are then concatenated along the channel axis, creating a rich representation of the input data at different levels of detail. This multi-scale information is invaluable for semantic segmentation tasks, as it helps the model make contextually informed decisions about object boundaries and features. Mathematical representation of SPP block given below:

Given input tensor **X** with dimensions (*H*, *W*, *C*), and considering a single pooling level. Perform max pooling with a pooling window size of *p* × *p*:

$$\begin{array}{c} Z_{p}\left(i,j,c\right)={\text{max}}_{a,b}\;X\left(p\cdot i+a,p\cdot j+b,c\right),\;\text{for}\;0\leq a,b<p \end{array}$$

Apply a 1x1 convolution to reduce the number of channels to 256:

$$\begin{array}{c} Z_{p}\left(i,j,f\right)=\sum_{c}\;W_{p}\left(f,c\right)\cdot Z_{p}\left(i,j,c\right) \end{array}$$

Upsample the feature map to the original size:

$$\begin{array}{c} Z_{p}\left(i,j,f\right)=Z_{p}(\frac{i}{p},\frac{j}{p},f) \end{array}$$

Concatenate the upsampled feature maps along the channel axis to form the final SPP output:

$$\begin{array}{c} Y\left(i,j,f\right)=[Z_{p}\left(i,j,f_{1}\right),Z_{1}\left(i,j,f_{2}\right),Z_{2}\left(i,j,f_{3}\right),...] \end{array}$$

Where, *H* and *W* represents the height and width of the input tensor. *C* represents the number of channels. *p* represents the pool size (a positive integer). *i* and *j* represent the spatial coordinates within the tensor. *c* and *f* represents the channel and filter index respectively. **W**_**p**_ represents the weights for the 1x1 convolution.

### 3.4 Fusion block

The Fusion Block plays a pivotal role in combining feature maps from multiple sources within the G-Net architecture. It takes a list of input tensors and concatenates them along the channel axis to create a fused input tensor. This fused input tensor is then passed through a convolutional layer with a specified number of filters, kernel size, activation function, padding, and kernel initializer. This block integrates network information and feature extraction methods, enhancing semantic segmentation and accuracy by capturing complex patterns and relationships, especially in scenarios requiring multiple levels of abstraction.

The Fusion Block starts with the concatenation of input tensors **X**_1_, **X**_2_, …, **X**_*n*_ along the channel axis to create a fused input feature map **F**:

$$\begin{array}{c} F=\text{Concatenate}(X_{1},X_{2},\ldots ,X_{n}) \end{array}$$

Then, a 2D convolution is applied to the fused input feature map **F**, resulting in the output feature map **Y**. The convolution can be represented as:

$$\begin{array}{c} Y=\text{Conv2D}(F,\text{filters},\text{kernel\_size},\text{activation},\text{padding},\text{kernel\_initializer}) \end{array}$$

Where, **F** is the fused input feature map. filters is the number of filters in the convolution. kernel_size denotes the size of the convolutional kernel. activation specifies the activation function applied to the output. padding is the padding method. kernel_initializer is the method for initializing the kernel weights.

Tables 1 and 2 provide the feature extraction procedure, the integration of distinct blocks, and the description and reasoning for each block. Integration and Feature Extraction process involves processing the input tensor through SE, multi-scale feature aggregation through the SPP block, feature fusion using the Fusion Block, feature extraction through the Convolutional Block, and attention mechanism through the SA Block. The tensor is then processed to adjust channel-wise features, capture contextual information at various scales, and refine features through two convolutional layers. The output is then processed to refine the feature map based on computed attention weights.

**Table 1:** Description and rationale of various blocks.

Function	Description	Rationale
SE	The process involves global average pooling, followed by two dense layers with ReLU and sigmoid activations. Finally, reshaping occurs, and the result is multiplied with the input tensor.	It improves channel interdependencies by dynamically recalibrating feature maps.
SPP	Max pooling is performed at various scales, followed by 1x1 convolutions and upsampling. The resulting features are then concatenated.	It captures and integrates spatial information across multiple scales, enhancing the feature maps.
Fusion Block	It combines multiple input tensors along the channel axis and then applies a convolutional layer.	This process integrates features from various stages or sources, enhancing the overall feature representation.
Conv Block	A common architectural unit for feature extraction and transformation consists of two successive convolutional layers. These layers are defined by specific filters, kernel sizes, activation functions, and padding.	Standard framework for identifying and transforming features.
SA	A 1x1 convolution with sigmoid activation generates attention weights, which are subsequently multiplied with the input tensor.	This process captures long-range dependencies and enhances the feature map by emphasizing important regions.

**Table 2:** Purpose, integration mechanism, and benefits of G-Net.

Block	Purpose	Description	Integration Mechanism	Benefits
Conv Block	Feature extraction and refinement	Sequential Convolutional Feature Extraction: Captures hierarchical patterns and spatial information.	The First Conv layer extracts initial features. Second Conv layer further refines the feature maps.	Extracts detailed spatial characteristics. Layering convolutions to enhance feature extraction depth.
SE	Channel recalibration	Adaptive Recalibration of Channel-wise Feature Responses: Adjusts feature responses for each channel dynamically. Enhances relevant information while suppressing noise.	Global Average Pooling aggregates each channel into a single value. The first layer of Dense Layers reduces dimensionality, while the second layer restores it. The output is reshaped and multiplied by the input tensor.	Strengthens connections between channels and improves the model’s capacity to prioritize the important features.
SA	Focus on important features	Long-range Dependency Capture by Focusing on Important Features: Considers distant relationships between features. Prioritizes relevant features for better context understanding.	Attention Weights step involves applying Conv2D to compute attention weights. After obtaining the attention weights, the input tensor is multiplied element-wise by these weights.	Emphasizes essential characteristics and enhances the model’s capacity to grasp contextual details.
SPP	Multi-scale feature extraction	Multi-scale Feature Extraction with Different Pooling Sizes: Extracts features at various scales using different pooling operations. Captures both fine-grained and global information.	Utilizes MaxPooling2D with different pool sizes. Then, applies Conv to each pooled feature. Upsamples it by Resizing each feature to match the input size and combines the original and upsampled features.	Collects contextual details across various levels. Manages input images with varying dimensions.
Fusion Block	Feature integration	Unified Feature Representation from Multiple Input Tensors: Combines features from different input sources into a cohesive representation. Creates a unified view of the data for downstream tasks.	Concatenates by Combining multiple input tensors along the channel dimension. Applies Conv on the fused input for feature extraction.	Combines data from various sources. Improves the feature representation by combining diverse perspectives.

### 3.5 Impact of our framework

Breakdown of each module’s’s operation to understand the functionality of the architecture. The model starts by applying ReLU activation and two convolutional layers with 64 filters to the input image through an initial convolutional block. Next, a SE module with global average pooling, dense layers with ReLU and sigmoid activations, and a reshaping step that multiplies the SE output by the input tensor to enhance significant features are applied to this output. Max-pooling is the procedure that comes after this to minimize spatial dimensions. The second and third convolutional blocks which use 128 and 256 filters, respectively are used in the same way. SE modules come after each block. Following the third block, a custom SPP layer pools features at several scales and concatenates them to generate a rich representation. SA mechanism then captures long-range dependencies. Upsampling the SPP layer’s output and utilizing fusion blocks to combine it with matching features from previous convolutional blocks constitute the decoder phase. Every fusion block applies a convolutional layer to combine the inputs after concatenating them. In order to ensure correct segmentation, the model employs a 1x1 convolutional layer with 4 filters and softmax activation to generate a segmentation mask. This allows for the detailed capture of features and their efficient combination. This architecture effectively combines SE, SPP, Fusion, SA block, convolutional layers to capture both local and global contextual information, enhance feature dependencies, and maintain fine spatial details, contributing to its proficiency in semantic segmentation.

## 4 Results and discussion

This section presents comprehensive experimental results demonstrating G-Net’s performance compared to other state-of-the-art models and the traditional U-Net. In order to build and train models for a variety of machine learning tasks, the system makes use of a PowerEdge R740 server equipped with an INTEL XEON Silver 4208 processor, Tesla V100 GPU, 128 GB DDR4 RAM, TensorFlow deep learning framework, and Keras API.

### 4.1 Dataset description

In our investigation using a typical Kaggle dataset, the data distribution for partitioning was as follows: Training: 425 images, Validation: 125 images, Testing: 75 images. Details of the data description are displayed in Table 3. Our initial approach involved partitioning the dataset at the slice level to maximize data utilization and maintain a balanced dataset. This method facilitated a comprehensive training phase, accounting for variations in tumor appearance across different slices. We ensured that all slices were exclusively assigned to either the training or testing set. Importantly, the consistent performance across both partitioning strategies reaffirms the generalizability and robustness of G-Net. Notably, the training and testing sets do not overlap with slices from the same patient. This design choice ensures that improvements in performance, particularly in dice scores for tumor regions, are not influenced by shared characteristics among slices from the set. Thus, our partitioning technique is appropriate and does not compromise the validity of our performance assessment.

**Table 3:** Description of dataset.

Hyperparameter	Description
Dataset	BraTS2020
Dataset source	Kaggle
Data Partitioning	Random split
Image size	224 × 224 × 1 × 150
Optimizer	Adam
Learning Rate	0.001
Activation Function	Relu
Overall no. of subjects and no. of images in each subject	625 and 5
Modality types	FLAIR, T1, T1ce, T2 and Seg
Segment Classes Types	Non-tumor, necrosis, edema, enhancing and non-enhancing tumor

### 4.2 Model and parameters training approach

**Fig 3:**
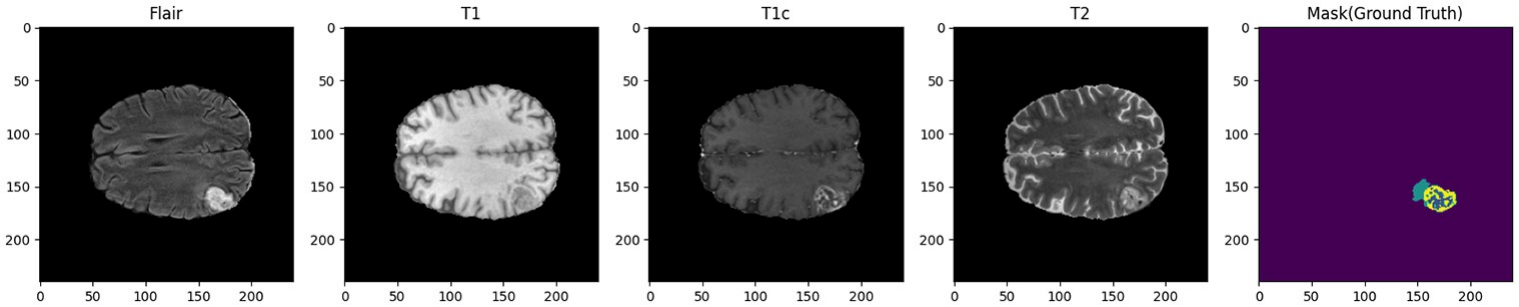
MRI sequences as 2D sections.

The G-Net model is trained with classified training data and a categorical cross-entropy loss function. Training specifics, like optimizer selection, learning rate, and information, are frequently explained in this section. The model proposed in this work is independently trained and validated four times using distinct MRI sequences. The BRATS 2020 datasets are available to the public, and they are used to assess the proposed framework. A patient receives 155 slices, each measuring 240 x 240 x 1 x 155, in an MRI scan. The BRATS dataset contained comparable T1, T1c, T2, and FLAIR MRI sequences from patients who had either high-grade or low-grade gliomas associated data sample shown in Fig 3. Each case was individually categorized into subgroups for peritumoral edema, enhancing tumor, and non-enhancing tumor core using the same labeling procedure.

### 4.3 Evaluation metrics

The following are then used to calculate the model’s accuracy, precision, and Eqs 1 and 2 are used for sensitivity and specificity based on the segmented ground truth of the tumor portion as provided by the MRI.

Sensitivity is calculated using Eq (1), where the cardinalities of sets *X* and *Y* are denoted as |*X*| and |*Y*| respectively. *G*_1_ represents the proportion of tumor regions in the ground truth images, and *X*_1_ represents tumor regions that were predicted by the model.

$$\begin{array}{c} \text{Sensitivity}\left(X,G\right)=\frac{|X_{1}\cap G_{1}|}{|G_{1}|} \end{array}$$ (1)

Specificity is calculated using Eq (2), where *G*_0_ represents non-tumor tissue regions of the ground truth, and *X*_0_ represents the non-tumor tissue regions predicted by the model.

$$\begin{array}{c} \text{Specificity}\left(X,G\right)=\frac{|X_{0}\cap G_{0}|}{|G_{0}|} \end{array}$$ (2)

$$\begin{array}{c} \text{Tversky}\;\text{Loss}\left(y_{\text{true}},y_{\text{pred}}\right)=1-\frac{\text{TP}+\text{smooth}}{\text{TP}+\alpha \cdot \text{FN}+\beta \cdot \text{FP}+\text{smooth}} \end{array}$$ (3)

Tversky Loss calculated with Eq (3) where, TP is the sum of true positives, FN is the sum of false negatives, FP is the sum of false positives. *α* and *β* are weights that control the penalties for false negatives and false positives, respectively, smooth is a small constant to avoid division by zero.

$$\begin{array}{c} \text{Boundary}\;\text{Loss}\left(y_{\text{true}},y_{\text{pred}}\right)=\text{Boundary}\;\text{Weight}\cdot \text{Mean}(|\text{Sobel}\left(y_{\text{true}}\right)-\text{Sobel}\left(y_{\text{pred}}\right)|) \end{array}$$ (4)

The boundary loss Eq (4) is computed using Sobel edge detection applied to the images *y*, and the mean absolute difference between the edges of the true and predicted images, scaled by a factor called boundary_weight.

In the results section of our research paper, we are pleased to present exceptionally high-performance metrics achieved after training our model for 30 epochs, firmly establishing the effectiveness of our approach. During this 30-epoch experiment, our model consistently achieved accuracy rates surpassing 99.42%, accompanied by a remarkably low loss value of 0.0165, demonstrating the model’s robust predictive capabilities and its proficiency in error minimization. Furthermore, precision scores consistently exceeded 99.50%, affirming the model’s ability to accurately identify positive cases while maintaining minimal false positives. Sensitivity consistently surpassed 99.26%, signifying the model’s capacity to capture a substantial proportion of true positive cases. Additionally, specificity exceeded 99.83%, showcasing its competence in effectively distinguishing between negative and positive instances. The 18th epoch of training was halted to prevent overfitting and optimize efficiency. The EarlyStopping callback was used to monitor validation loss, and if no improvement was observed for five consecutive epochs, training was terminated. This decision highlighted the importance of closely monitoring and fine-tuning the training process, especially when additional epochs may not significantly enhance performance. The study continued training until the 50th epoch after implementing early stopping at the 18th epoch to explore the model’s performance, ensure stability, experiment with hyperparameters, and meet specific training objectives, justifying the extended training duration for comprehensive assessment and optimization. Values obtained of epoch are given in Table 4.

**Table 4:** Performance comparison of G-Net for different epochs.

Performance Matrix	Training of G-Net		Val of G-Net	
	Epoch 18	Epoch 50	Epoch 18	Epoch 50
**Accuracy**	99.37	99.42	95.53	97.81
**Loss**	0.0186	0.0165	0.1578	0.0607
**Precision**	99.43	99.50	97.10	98.10
**Sensitivity**	99.22	99.26	94.82	97.53
**Specificity**	99.80	99.83	99.05	99.36
**Tversky loss**	0.0094	0.0094	0.0532	0.0274
**Boundary loss**	0.0261	0.0246	0.0752	0.0439

**Fig 4:**
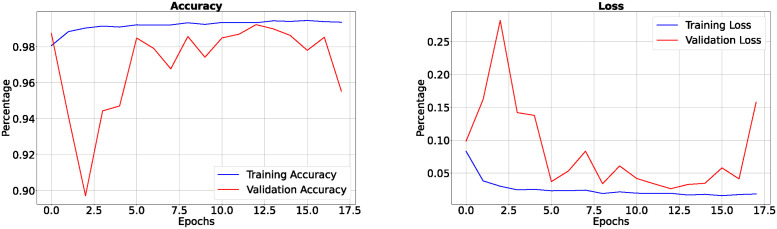
Accuracy, loss when trained for 18 epoch.

**Fig 5:**
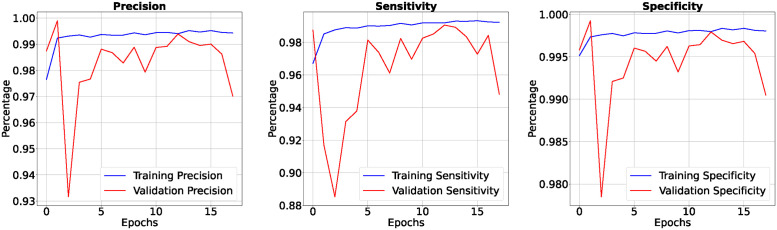
Precision, sensitivity and specificity when trained for 18 epoch.

**Fig 6:**
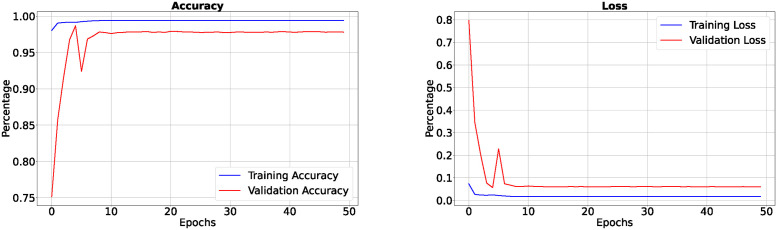
Accuracy, loss when trained for 50 epoch.

**Fig 7:**
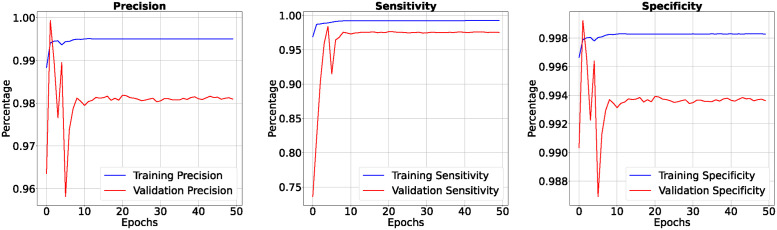
Precision, sensitivity and specificity when trained for 50 epoch.

**Fig 8:**
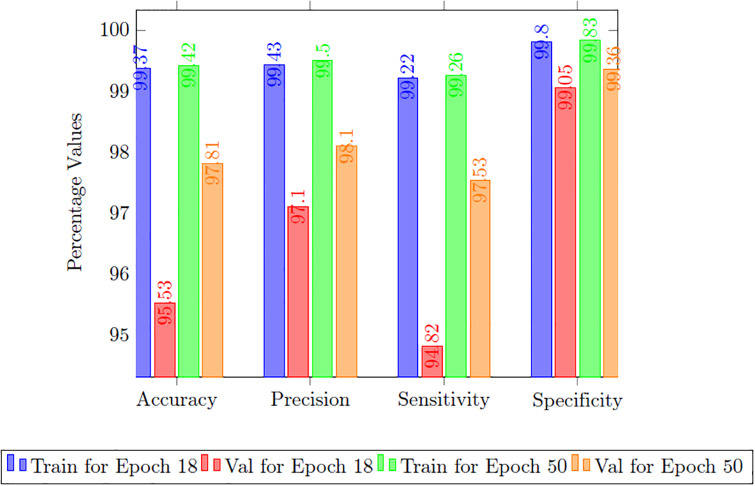
Performance comparison of accuracy, precision, sensitivity, specificity for 18 and 50 epochs.

**Fig 9:**
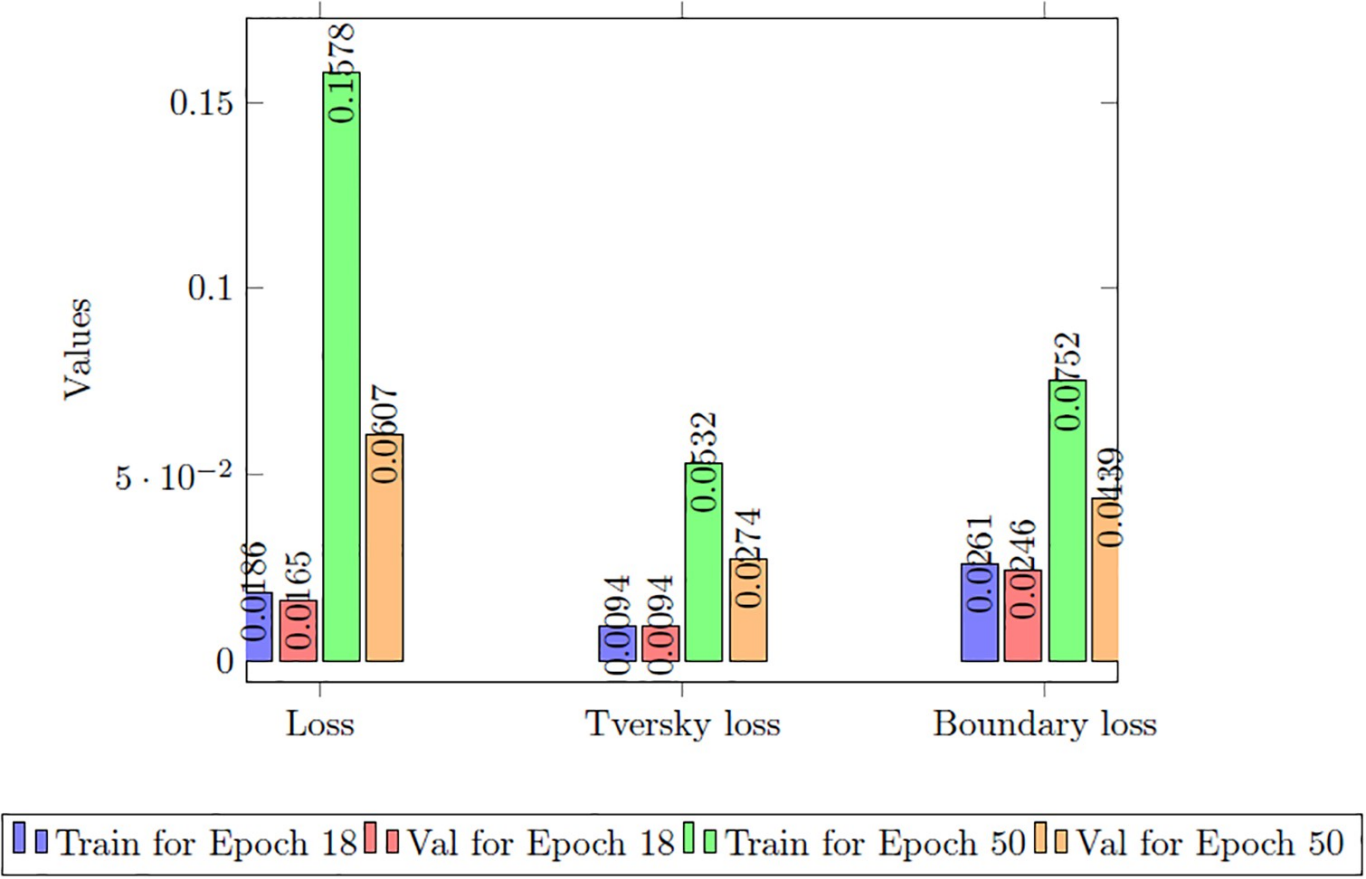
Performance comparison of loss, tversky loss, boundary loss for 18 and 50 epochs.

**Fig 10:**
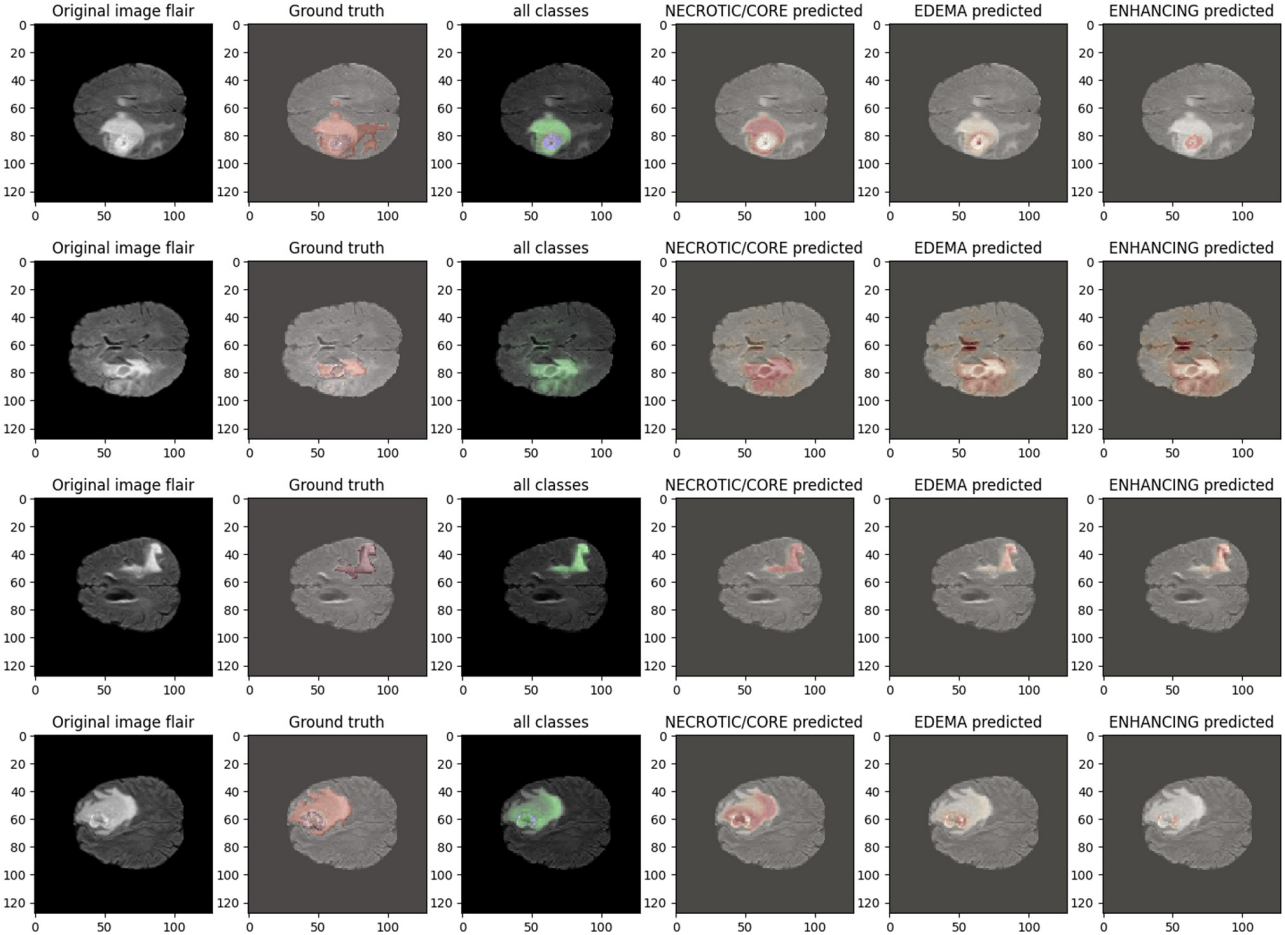
Illustration presented below displays the qualitative outcomes of the G-Net model applied to BraTS2020.

**Fig 11:**
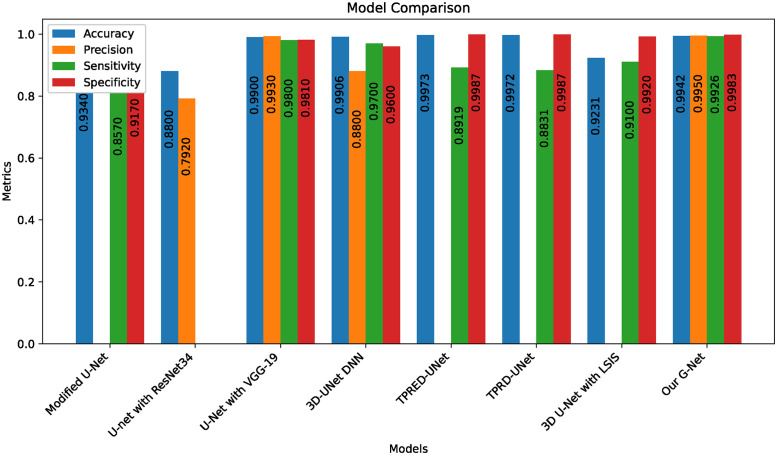
Following visualization showcases the results of the G-Net over existing models ([5, 10, 11, 18, 42, 44]) on BraTS2020.

In the 50-epoch experiment, our model consistently delivered outstanding results, achieving accuracy rates consistently surpassing 99.42%, maintaining low loss values of 0.0165, and demonstrating precision scores of 99.50%, effectively minimizing false positives. Our research showcased a remarkable sensitivity exceeding 99.26%, underscoring the model’s proficiency in identifying true positive cases, while consistently upholding a specificity of 99.83%, demonstrating its excellence in distinguishing negative instances. The Tversky loss was calculated to be 0.0094, and the boundary loss was measured at 0.0246. After 50 epochs, we obtained a dice coefficient of 0.7034 for edema and 0.7091 for enhancing. The outcomes of Accuracy and loss when trained for 18 epoch and Precision, Sensitivity, Specificity for Epoch 18 are presented in Figs 4 and 5. Epoch 50 are presented in Figs 6 and 7 respectively. Notably, these high-performance metrics not only remained stable but also exhibited marginal improvement during the 50-epoch experiment, further highlighting the robustness and reliability of our methodology over extended training periods. This graph shown in Figs 8 and 9 displays ML model’s performance metrics at critical epochs 18 and 50, showing significant enhancement over time, with lower loss values and improved accuracy and classification metrics. The better outcomes are demonstrated by the precision with which tumor classifications and specific core, whole, and augmenting tumors can be distinguished, as seen in columns 4, 5, and 6 of the images. The most recent techniques are used to compare the DSC readings for 2020 and 2021 brats data for Whole Tumor (WT), Tumor Core (TC), and Enhanced Tumor (ET) in Tables 5 and 6. The descriptive results of using the G-Net model with BraTS2020 are shown in the Fig 10. The most significant findings enumerate the results highlighted in Table 7 and Fig 11 shows the qualitative outcomes of the G-Net model applied to BraTS2020.

**Table 5:** Performance comparisons of Subclass tumor using Brats 2020.

Algorithms	WT	TC	ET
**Self-calibrated attention U-Net** [28]	0.905	0.821	0.781
**Attention-based CNN with U-Net** [10]	0.90	0.86	0.83
**Double attention U-Net** [27]	0.8912	0.8427	0.7915
**AD-Net** [8]	0.872	0.823	0.803
**dResU-Net** [12]	0.8660	0.8004	0.8357
**Residual Spatial Pyramid Pooling-powered U-Net** [28]	0.904	0.880	0.845
**Deep multi-task learning with multi-depth fusion module** [29]	0.860	0.772	0.700
**Convolutional block attention V-Net** [30]	0.876	0.769	0.670
**AGSE-VNet** [31]	0.68	0.85	0.70
**CNN** [32]	0.890	0.814	0.785
**Bridged U-Net ASPP** [34]	0.9073	0.8159	0.78
**G-Net**	0.9782	0.8294	0.8254

**Table 6:** Performance comparisons of subclass tumor using Brats 2021.

Algorithm	WT	TC	ET
**dResU-Net** [12]	0.8601	0.8400	0.8221
**Residual Spatial Pyramid Pooling-powered U-Net (2 SPP)** [13]	0.886	0.876	0.843
**Residual U-Net (1 SPP)** [13]	0.887	0.879	0.842
**U-Net with Attention block** [33]	0.879	0.819	0.793
**Bridged U-Net_ASPP (var-1)** [34]	0.9187	0.8594	0.8434
**Bridged U-Net_ASPP (var-2)** [34]	0.9251	0.8658	0.8395
**G-Net**	0.9643	0.8445	0.8512

**Table 7:** Comparison of G-Net with existing techniques on BraTS2020.

Model	Accuracy	Loss	Precision	Sensitivity	Specificity
Modified U-Net [5]	0.934	0.26		0.857	0.917
U-Net with ResNet34 [11]	0.88	0.208	0.792		
U-Net with VGG-19 [10]	0.99	0.054	0.993	0.98	0.981
UNet DNN [18]	0.9906	0.17	0.88	0.97	0.96
LSIS [42]	0.9231			0.91	0.992
Deep CNN and U-Net [43]	0.987	0.066	0.984	0.987	0.996
Our G-Net	0.9942	0.0165	0.9950	0.9926	0.9983

## 5 Discussion and future direction

To fully realize the potential of this model in clinical settings, several critical aspects must be considered. First and foremost, regulatory approval is essential. Rigorous testing and validation on diverse datasets are necessary to demonstrate the model’s reliability and safety as a medical device. Healthcare professionals need proper training to interpret and utilize the segmentation results effectively, including understanding the model’s limitations and potential sources of error. Interoperability is crucial—ensuring that the model can seamlessly integrate with various imaging modalities and healthcare IT systems. Continuous improvement mechanisms, such as incorporating feedback from clinical use and updating the model with new data, are vital. Finally, conducting thorough cost-benefit analyses will demonstrate the economic advantages of adopting the model, including time savings, reduced diagnostic errors, and improved patient outcomes. By addressing these aspects, we can successfully integrate the model into clinical practice, enhancing medical diagnostics and patient care. The G-Net Model Integration and Performance Analysis Table 8 highlights the model’s limitations and suggests future approaches, with a special emphasis on how well the model scales to accommodate different tumor categories.

**Table 8:** Model integration and performance analysis of G-Net.

Aspect	Details
**Strengthening the Model**	Integration of SE blocks enhances feature recalibration, SA mechanisms improve context awareness, and SPP effectively handles scale variations. This combined approach significantly enhances segmentation accuracy and robustness.
**Performance Outcome**	Initial findings indicate enhanced segmentation metrics, including increased accuracy, precision, and sensitivity, resulting from improved feature extraction and fusion mechanisms.
**Challenges Faced**	The model’s added layers (SE, SA, SPP) increase computational complexity. These additional layers lead to prolonged training times. It’s necessary to find optimal settings for the new blocks.
**Measured and Evaluated**	We employed metrics such as accuracy, loss, precision, sensitivity, and specificity. Our evaluation aimed to assess the impact of each block on segmentation performance.
**Model Discussion**	The combination of SE blocks, SA, and SPP significantly enhances feature extraction and segmentation capabilities, leading to improved metrics like accuracy, precision, and sensitivity.
**Limitations**	The model integrates SE blocks for feature map recalibration, SA to capture long-range dependencies, and SPP to effectively handle multi-scale features.
**Impact of All Blocks**	SE Enhanced feature adjustment and increased network capacity. Also enhances feature recalibration and boosts relevant features. SA Increased attention to critical features and distant features. It captures long-range dependencies, enhancing context awareness. SPP Improved management of tumor size variations. It handles multi-scale features, improving segmentation across varying scales. Fusion Blocks Maintained fine details for precise segmentation.
**Future Directions**	**Model Scalability:** Investigate the model’s performance on different types of tumors. Assess its generalization to diverse tumor datasets. **Optimization:** Implement techniques to reduce computational complexity during training. Focus on faster convergence without sacrificing performance. **Transfer Learning:** Utilize pre-trained weights from existing models to accelerate training on new tumor datasets. Leverage knowledge learned from related tasks for improved efficiency.

The model’s future directions include enhancing its generalizability by testing it on various tumor types beyond the current dataset, developing lightweight architectures to reduce computational overhead, addressing class imbalances in multi-class segmentation tasks to ensure fair representation of all tumor classes, and optimizing the model for real-time performance in clinical settings for timely decision-making and patient care. These advancements will help improve the model’s applicability and efficiency in practical applications.

## 6 Conclusion

The research improves the appearance of target images for better patient care by introducing an automated technique for segmenting and measuring brain tumors using the BRATS 2020 MRI dataset. It has been shown that for the segmentation of brain tumors, the proposed G-Net outperformed the U-Net and existing state of art techniques. The quantitative evaluations showed that the proposed technique produced satisfactory outcomes when erroneously segmenting brain tumors. The method also showed how the segmentation process and outcomes might be improved by using the enhancement phase as a prior to treatment stage. This technique helps identify brain tumors early on, which can lead to successful treatment and even save lives. It improves diagnostic precision and lowers death rates by generalizing network networks, predicting tumor aggressiveness, and optimizing computational resources. G-net model for segmenting brain tumors from MRI images is suggested in this research. This technique extracts ROIs (tumor areas in brain MRI) from each unique MRI sequence it gets as training data. Input are normalized and rescaled into single 128 128 images in order to reduce computational expense and boost efficiency. The field of BTS requires enhancement of models like G-Net, domain adaptation, real-time integration with medical imaging systems, making AI-driven segmentation more understandable, personalizing models for individual patient characteristics, dealing with data scarcity through data augmentation, collaboration with medical experts, rigorous clinical validation, and scalability and accessibility through subsampling. The seamless integration of the G-Net model into clinical protocols ensures its practical applicability and effectiveness. It will facilitate real-time tumor segmentation, aiding in diagnosis and treatment decisions. Rigorous performance assessments will occur in clinical environments, with invaluable input from medical experts driving enhancements. G-Net’s scope will extend to encompass various medical imaging tasks, enhancing patient outcomes and diagnostic accuracy. Future research should investigate its integration within clinical workflows, assess its real-world performance, and explore its utility across imaging modalities such as PET, CT, and ultrasonography.
